# Distal bypass with a varicose vein graft for critical limb ischemia: report of a case

**DOI:** 10.1186/s40792-019-0755-x

**Published:** 2019-12-10

**Authors:** Atsushi Guntani, Ryosuke Yoshiga, Shinsuke Mii

**Affiliations:** Department of Vascular Surgery, Saiseikai Yahata General Hospital, 5-9-27 Haruno-machi, Yahatahigashi-ku, Kitakyushu, 805-8527 Japan

**Keywords:** Critical limb ischemia, Varicose vein, Bypass surgery, Limb salvage

## Abstract

**Background:**

A saphenous vein complicated with varicose veins is generally thought to be unsuitable for bypass grafting.

**Case presentation:**

A patient who developed sepsis due to lower limb gangrene was successfully treated by endovascular treatment and bypass surgery using a varicose vein graft. There were no complications, such as occlusion or aneurysm, of the varicose vein graft during the 2-year follow-up period.

**Conclusions:**

We herein report a case in which bypass surgery with a varicose vein graft was used to avoid major amputation of the lower limb, and the patient recovered markedly from sepsis. If there are no other appropriate autologous veins for revascularization of lower limb gangrene, a varicose vein graft may be useful as a conduit for bypass surgery at risk of graft infection.

## Background

It is generally recommended that varicose vein grafts not be used, and few prospective case studies have been reported, with evidence insufficient to draw conclusions at this point [1–4]. We successfully treated a patient with lower limb gangrene who received immediate inflow artery reconstruction by endovascular treatment and debridement of a necrotic foot and then underwent complete revascularization by bypass surgery using a saphenous vein graft complicated with varicose veins.

## Case presentation

**Fig. 1:**
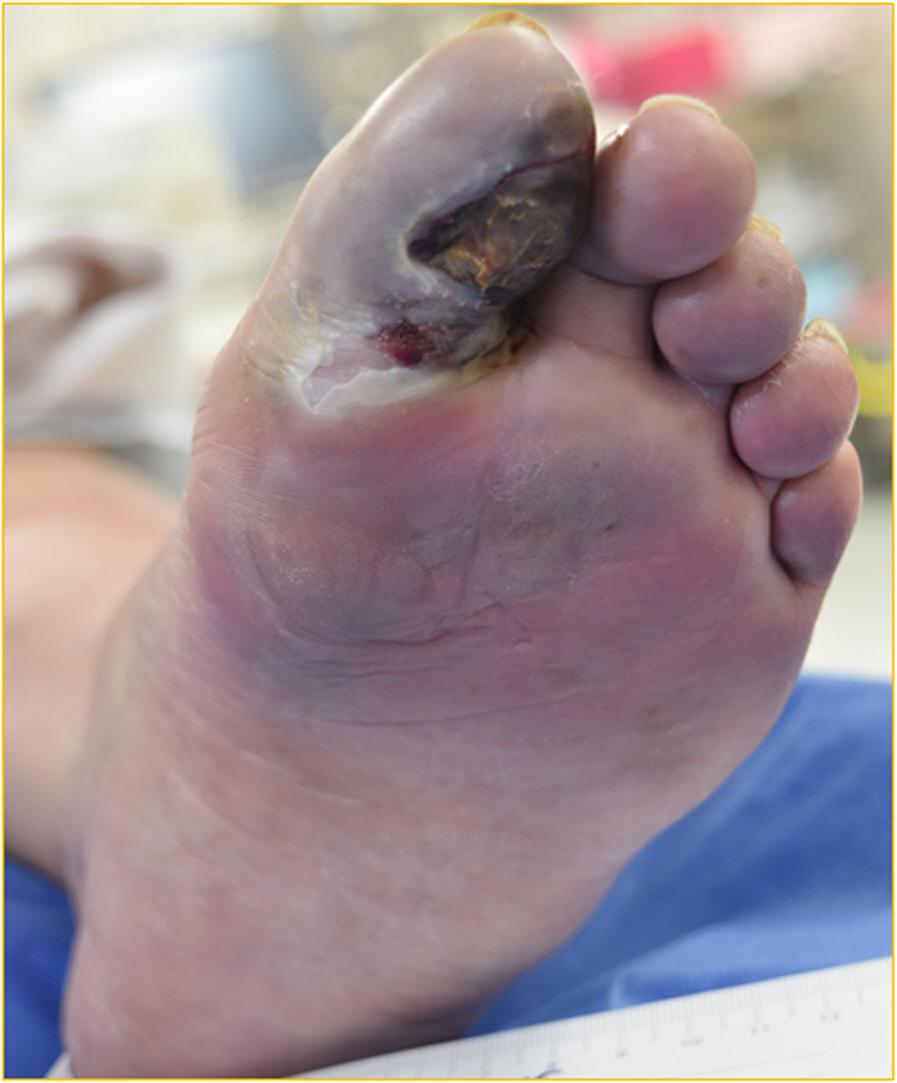
The picture on admission showed that the left foot was swollen red, and the first toe was gangrenous

An 80-year-old female patient was admitted for severe pain in the left foot. She had injured the sole of the left toes a few days before admission, and the area had become ulcerated. She had a history of diabetes, coronary artery disease, and renal disfunction that did not require dialysis; however, all of her diseases were being controlled with medication. On admission, the left foot was swollen red, and the first toe was gangrenous (Fig. 1). She had a fever of 39 °C with respiratory distress, and her level of consciousness was “delirium.”

A laboratory examination showed that the white blood cell (WBC) count and C-reactive protein (CRP) level were increased at 21900/μl and 28.51 mg/dl, respectively. The left lower limb pulse was not palpable, the ankle-brachial index (ABI) was 0.36, and the skin perfusion pressure (SPP) of the foot was 14 mmHg. A duplex scan showed no flow in the left external iliac artery (EIA) and a poor flow below the left femoral artery. We clinically diagnosed her with severely ischemic limbs with sepsis due to foot gangrene.

**Fig. 2:**
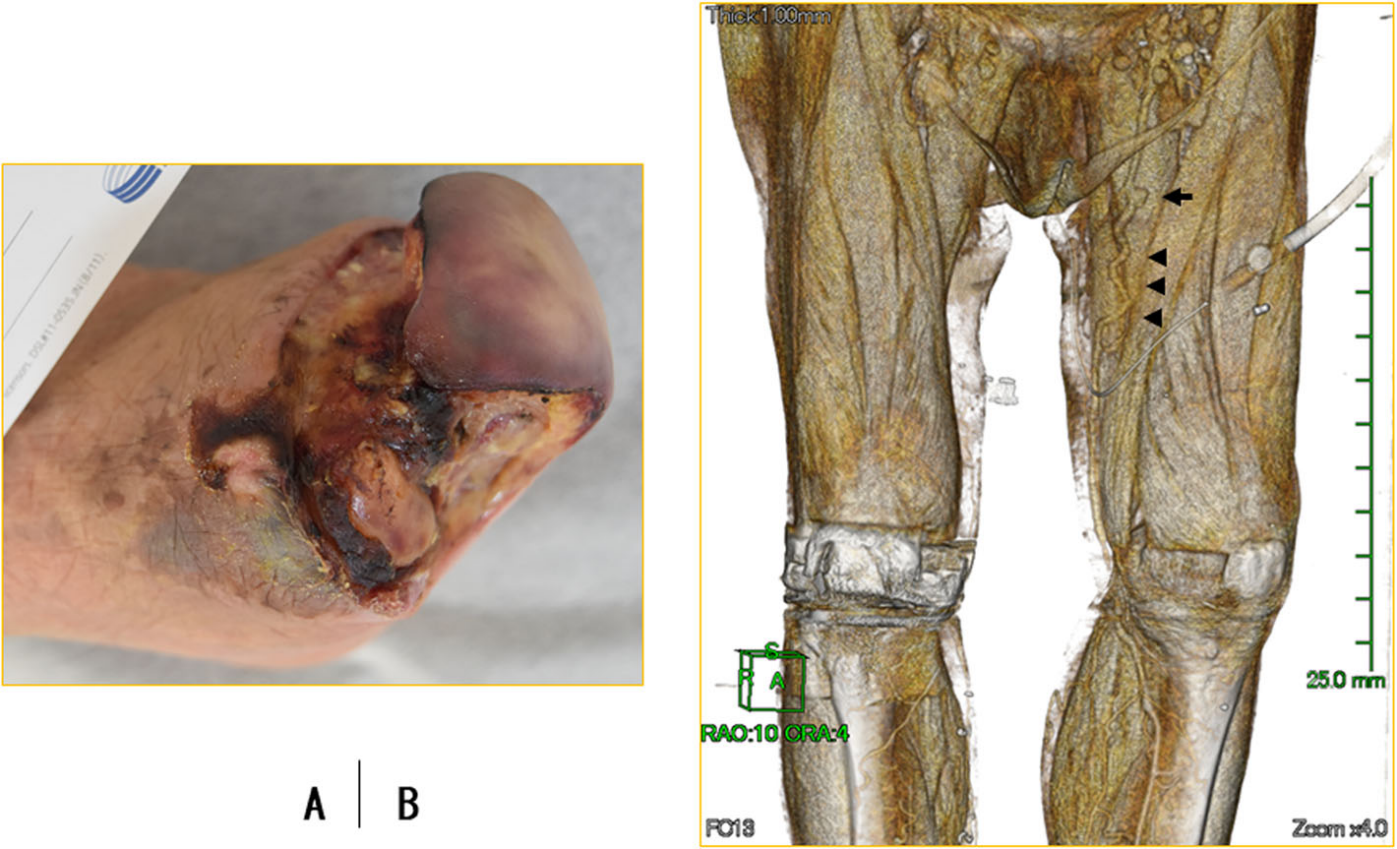
The stump became gradually necrotic after metatarsal amputation (**a**). Preoperative plain computed tomography showed that the left great saphenous vein was complicated with varicose veins (arrowheads) with thin vessel walls (arrow) (**b**)

Broad-spectrum antibiotics of meropenem and vancomycin were immediately administered, and the necrotic first toe was amputated under local anesthesia with pus discharged on the day after admission; methicillin-resistant *Staphylococcus aureus* was detected in culture of the pus. Subsequent angiography of the left lower limb revealed the presence of an occlusive lesion of the EIA and below the distal superficial femoral artery (SFA); however, the dorsal artery was enhanced via the collateral circulation. Endovascular angioplasty of the EIA was performed with a bare metal stent (SMART Control 8.0 mm × 100 mm; Cordis, Miami Lakes, FL, USA). The gangrenous area had gradually been confined to the toes. Three days later, metatarsal amputation was performed under sciatic nerve block anesthesia (Fig. 2a). At 10 days after admission, the fever decreased to 36 °C, and her level of consciousness recovered to a clear state. The WBC count and CRP level decreased to 13800/μl and 11.85 mg/dl, respectively.

Left femoro-dorsal artery bypass was performed under general anesthesia using the ipsilateral great saphenous vein in a reversed fashion 14 days after admission. However, ultrasonography to make a preoperative evaluation revealed that the left great saphenous vein was complicated with varicose veins (dilatated generally to about 6 mm or 8 mm in diameter, with some aneurysms measuring up to 10 mm in diameter), and there were no other appropriate autologous veins for femorotibial artery bypass. There was no contralateral great saphenous vein, probably due to her history of varicose vein surgery (details unknown). In addition, since the bilateral short saphenous veins and arm veins were small in diameter, bypass surgery without using a varicose vein graft was impossible. Therefore, we decided to use the varicose vein graft for the bypass in order to salvage the limb instead of a prosthetic graft at the risk of infection. When the vein graft was gently distended with saline containing heparin using a syringe for preparation, the saccular venous aneurysm was partially resected (Fig. 2b, arrow). The remaining tortuous and dilatated varicose vein graft was used at its full length without splicing (Fig. 2b, arrowheads).

**Fig. 3:**
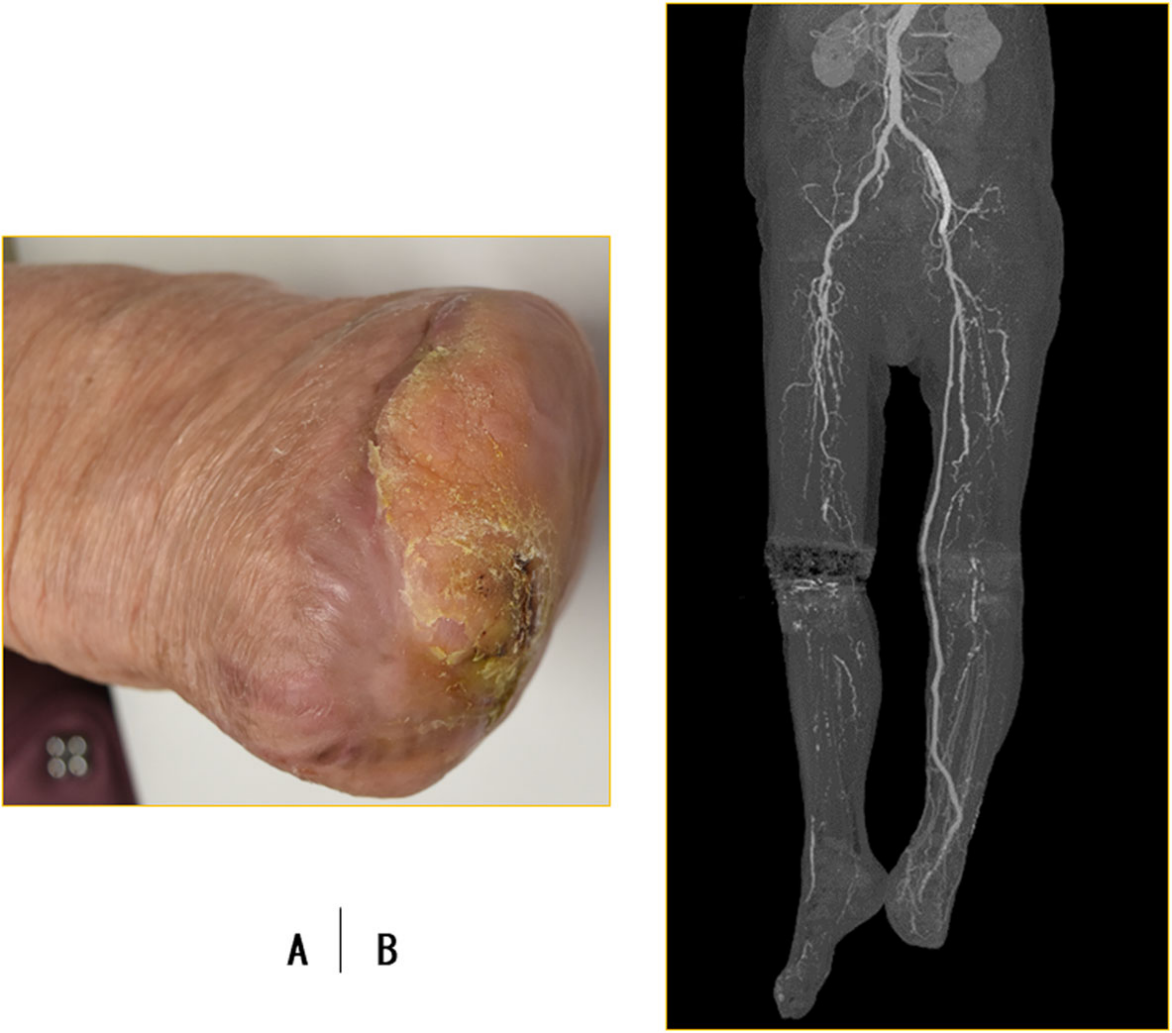
The stump of the amputated toes completely healed (**a**, 6 months after the bypass surgery). Follow-up contrast-enhanced computed tomography revealed that there were no complications, such as occlusion or aneurysm, of the varicose vein graft (**b**)

After the bypass surgery, the blood flow of the left lower limb improved significantly, and the ABI and SPP increased to 0.88 and 60 mmHg, respectively. Several debridement operations were required; finally, the stump of the amputated toes had completely healed by 6 months after the bypass surgery (Fig. 3a). The patient was followed regularly as an outpatient at intervals of 3 months for graft surveillance using duplex ultrasonography. However, even on follow-up computed tomography (CT), there were no signs of complications, such as occlusion or aneurysm of the varicose vein graft, during the 2-year follow-up period (Fig. 3b).

## Discussion

The immediate removal of the necrotic tissue and complete revascularization are believed to have led to the recovery from sepsis and limb salvage in the present case. In bypass surgery, varicose vein grafts played an important role in achieving complete revascularization. In general, a varicose vein graft is considered unsuitable for bypass surgery [1–4], but in our case, the graft remained patent for a long time and played an important role in limb salvage.

In previous studies, minor dilatation of varicose vein was found able to be corrected by plication, tuck stitching, or prosthetic reinforcement and was able to be used as an autologous vein bypass graft. However major extensive dilatation of varicose veins and unfavorable flow dynamics predispose these veins to aneurysm formation and rupture. Furthermore, varicose vein grafts are likely to undergo increased intimal hyperplasia and long-term risk of thrombosis compared with normal vein grafts when exposed to arterial pressure [5–11].

However, there is not enough evidence concerning the long-term outcomes of varicose vein grafts. In our case, the use of a prosthetic graft had to be avoided because of the risk of graft infection. Therefore, we resected part of the saphenous vein with the saccular venous aneurysm under compression with saline containing heparin at the preparation of the vein graft, although most of the tortuous and dilatated varicose vein graft was still used. No increased intimal hyperplasia or thrombosis of the graft has been observed in the follow-up period.

Ultrasonography is usually used for the prevalence of a saphenous vein graft and is the most important evaluation method on diagnosis of varicose vein; however, we additionally performed a new technique (three-dimensional [3D] venous detection) wherein the image was manually adjusted for the window width and level setting in order to visualize the saphenous veins using plain CT reconstructed in 3D (Virtual Place; AZE Inc., Tokyo, Japan) (Fig. 2b). This method makes it possible to perform 3D visualization of the saphenous veins of the entire lower limb and determine whether or not the length of the saphenous veins is sufficient for a bypass graft. However, since the saphenous veins are evaluated under hydrostatic pressure using CT images taken in the supine position, compression with saline at the preparation of the vein graft during the operation is the best way to assess the venous changes when exposed to arterial pressure. Saphenous veins with partial eccentric venous aneurysms can be used as a graft by ligating or stitching the aneurysms; however, extensive dilatated in fusiform varicose veins should be excised if the length of the saphenous veins is sufficient for the bypass surgery. In the present case, no aneurysms were found to have developed in the varicose vein graft on 2-year follow-up CT; however, since the changes in the vein grafts under arterial pressure are not clear, further follow-up will be necessary.

## Conclusion

We herein report a rare case of sepsis developing due to lower limb gangrene that was successfully treated by endovascular treatment and bypass surgery using a varicose vein graft.

## Data Availability

All datasets supporting the conclusions of this article are included in this published article.

## Abbreviations

WBC: White blood cell
CRP: C-reactive protein
ABI: Ankle–brachial index
SPP: Skin perfusion pressure
EIA: External iliac artery
SFA: Superficial femoral artery
CT: Computed tomography
3D: Three-dimensional

## References

[1] Barker SGE, Hancock JH, Baskerville PA (1996). True aneurysms of infrainguinal vein bypass grafts: the need for active, not passive management. Eur J Vasc Endovasc Surg.

[2] Libertiny G., Galland R.B. (2001). Thrombus Formation Distal to Retained Valve Cusp in Varicose Segment of in situ Vein Graft: an Indication for Vein Reversal. European Journal of Vascular and Endovascular Surgery.

[3] Mun YS, Cho BS, Jang JH, Lee MS, Kwon OS (2016). Femoropopliteal Bypass with varicose greater saphenous vein. Int J Angiol.

[4] Cassina PC, Hailemariam S, Schmid RA, Hauser M (1998). Infrainguinal aneurysm formation in arterialized autologous saphenous vein grafts. J Vasc Surg.

[5] Charles AK, Gresham GA (1993). Histopathological changes in venous grafts and in varicose and non-varicose veins. J Clin Pathol.

[6] Davies MG, Hagen PO (1995). Pathophysiology of vein graft failure: a review. Eur J Vasc Endovasc Surg.

[7] Wilson YG (1998). Vein quality in infrainguinal revascularisation: assessment by angioscopy and histology. Ann R Coll Surg Engl.

[8] Soury P, Peillon C, Watelet J, Planet M, Plissonnier D, Gallo GD (1999). Prosthetic reinforcement of varicose saphenous vein grafts for infrainguinal bypass. Ann Vasc Surg.

[9] Hynes N, Mahendran B, Tawfik S, Sultan S (2006). Reinforced long saphenous vein bypass graft for infrainguinal reconstruction procedures: Case Series and Literature Review. Vascular.

[10] Iida H, Sunazawa T (2011). Self-Covered technique for a varix on a saphenous vein graft. Ann Thorac Surg.

[11] Cohn JD, Korver KF (2006). Selection of saphenous vein conduit in varicose vein disease. Ann Thorac Surg.

